# Obese elderly with diabetes experience more pain and reduced quality of life compared to obese elderly with hypertension

**Published:** 2020-05-12

**Authors:** Manuela Sodré Cabral, Nuno Manuel Frade de Sousa, Ramires Alsamir Tibana, Thiago dos Santos Rosa, Alessandro de Oliveira Silva, Silvana Schwerz Funghetto, Fabrício Azevedo Voltarelli, Milton Rocha de Moraes, Guilherme Borges Pereira, Gislane Ferreira de Melo, James W. Navalta, Jonato Prestes

**Affiliations:** ^1^Graduation Program in Gerontology and Graduation Program in Physical Education, Catholic University of Brasilia, Brasilia, Brazil; ^2^Laboratory of Exercise Physiology, Faculty Estacio of Vitoria, Vitoria, Brazil; ^3^Department of Physical Education, Federal University of Mato Grosso, Cuiaba, Brazil; ^4^Department of Physical Education, Faculty of Education and Health Sciences, University Center of Brasilia (UniCeUB), Brasília, Brazil; ^5^Graduation Program in Health Sciences, University of Brasilia (UnB), Brasilia, Brazil; ^6^Department of Physiological Sciences, Faculty of Education and Health Sciences, Federal University of São Carlos, São Carlos, Brazil; ^7^Department of Kinesiology and Nutrition Sciences, University of Nevada, Las Vegas, Las Vegas, Nevada, USA

**Keywords:** aging, diabetes mellitus, hypertension, muscle strength, obesity

## Abstract

**Background and Aim:**

Growth of elderly population is a worldwide phenomenon that impacts public health. The objective of this study was to compare the pain levels, strength, and quality of life among elderly obese with diabetes or hypertension.

**Materials and Methods:**

The study cohort comprised 52 obese elderly subjects with hypertension (n = 35) and diabetes (n = 17). The parameters measured were anthropometric features, handgrip strength, visual analog scale for pain, and quality of life using the World Health Organization questionnaire.

**Results:**

The level of pain reported by obese hypertensive elderly subjects (5.3 ± 3.4) was lower than reported by obese diabetic elderly subjects (7.4 ± 2.4). Obese hypertensive elderly scored higher on quality of life (sensory functioning and past, present, and future [PPF] activities) than obese diabetic elderly. No differences were observed for the other parameters. Strength, pain, anthropometrics, and hemodynamics were not correlated to quality of life.

**Conclusions:**

Obese elderly diabetics exhibit worse pain scores, sensorial abilities, and PPF activities than obese hypertensive elderly individuals.

**Relevance for Patients:**

The difference in pain and quality of life aspects between obese elderly individuals with hypertension and diabetes should be accounted for in health-care programs designed for these individuals.

## 1. Introduction

Gerontology is a multidisciplinary science that investigates different dimensions of the aging process and seeks to determine a wide array of knowledge surrounding the genesis of senescence and its deleterious effects [1-3]. Thus, gerontology offers useful information for healthy and quality aging [1-3].

Worldwide, there were 962 million in 2017 people aged 60 years or over in 2017. Their number is projected to grow to 2.1 billion in 2050, which provides evidence regarding the aging phenomena worldwide [4]. At present, Brazil presents an elevated and increasing prevalence of elderly individuals [5], estimated to grow to 64 million by the year 2050 [6] due to the demographic and epidemiologic transition that increases life expectancy in the aging population. This scenario is in response to a combination of factors, such decreased rates of fertility, decreased mortality, and improved health services and technology [5].

The aging process is characterized by increasing fragility, which prompts health-related initiatives and studies to protect elderly population on several fronts, including physical, mental, and social well-being [5,7,8]. The growing number of the elderly worldwide is associated with an increase in chronic non-communicable diseases (NCDs) [9]. Next to obesity, which is an important disease in the elderly, the NCDs with the highest impact on public health include cardiovascular diseases (including hypertension), cancer, diabetes, and chronic pulmonary diseases [10].

Obesity, hypertension, and diabetes in the elderly compromise functionality, resulting in debilitating conditions such as joint dysfunction and progressive loss of autonomy (AUT), with increasing chronic pain and a decrease in quality of life and dependence [11]. However, most studies compare the deleterious effects of aging in the elderly with and without a disease, while comparisons between different diseases are lacking [12-14]. Moreover, the increase in elderly individuals with NCD has brought more attention to the evaluation of quality of life, which has been accounted for in clinical studies [15-17]. Quality of life can be used to analyze several domains, from satisfaction with life to independence, control, and social and cognitive competences [15-17]. Investigation of the clinical condition in the elderly with distinct diseases could help to specify treatment and properly manage health.

Thus, the aim of this study was to compare the level of pain, muscle strength, and quality of life between elderly obese individuals with either hypertension or diabetes as comorbidity. The initial hypothesis is that obese elderly with diabetes present worse scores of quality of life and pain levels, without differences in muscle strength compared to obese elderly with hypertension.

## 2. Materials and Methods

### 2.1. Subjects

This was a transversal study involving 52 elderly subjects; 35 elderly obese with hypertension (68.3±6.4 years; 33.1±4.9 kg/m^2^) and 17 elderly obese with diabetes (68.6±5.5 years; 33.7±8.8 kg/m^2^) living in Barreiras – Bahia/Brazil. Obesity, hypertension, and diabetes were diagnosed by an experienced physician. Data were collected between February and March of 2018 in Basic Health Units. The inclusion criteria were as follows: Elderly people participating of the registration and monitoring system for hypertension and diabetes (Hiperdia) for at least 6 months before the research, age of ≥60 years, and signing the consent form to make part of the study. The study participants should not present with fibromyalgia, herniated disc, cancer, or any other disease that could increase pain, no use of analgesics or anti-inflammatory medications, recent surgery, and women should not be under hormonal replacement therapy. The present research was approved by the Local Ethics Committee for Human Use (protocol number 2.450.752).

### 2.2. Blood pressure measurement

Blood pressure measurements were obtained by the auscultatory method with cuffs adjusted to the size of the upper arm of the elderly according to the guidelines from the American Heart Association [14]. Three measures were taken with 1 min of rest between them, and the mean value of each subject was considered. Subjects were advised to seat calmly for 5 min, to empty their urinary bladder, to avoid exercise, smoking, alcoholic drinks, and coffee 24 h before the procedure, and not to eat 30 min before the measures. The elderly were also advised not to cross their legs, to maintain their feet on the ground, back in the chair, while the cuff was positioned in the arm at the level of the heart with palm up and slightly flexed elbow. The aneroid sphygmomanometer for obese adults was used (circumference of the arm cuff 35-51 cm; Premium, Brazil).

### 2.3. Blood glucose

Blood samples (25 μL) were collected from the fingertip earlobe after proper cleaning. Blood glucose concentration was determined by the electroenzymatic method (G-Tech Free Equipment, South Korea).

### 2.4. Anthropometrics

Body mass (to the nearest 0.1 kg) was measured in calibrated digital scale (Balmak-BKH-200 FAN, Brazil) and height (to the nearest 0.1 cm). Body mass index (BMI) was then calculated (body mass/height^2^). The BMI classification was according to the WHO (2000) as follows: BMI from 30 to 34 kg/m², Grade I obesity; BMI from 34 to 39.9 kg/m², Grade II obesity; and BMI ≥40 kg/m², Grade III obesity. All circumferences were obtained using non-elastic tape, and measurements were obtained in triplicate and averaged. Waist circumference was measured at the midpoint between the lower rib margin, and hip circumference was measured at the widest portion of the buttocks. Waist-to-hip ratio was calculated as WC divided by hip circumference.

### 2.5. Handgrip muscle strength

Handgrip strength was obtained by a manual handgrip hydraulic dynamometer (JAMAR, USA). Three measures on the right and left hand were obtained and the highest value was recorded. Elderly subjects were asked to keep their forearms in a neutral position, elbow fully extended, and standing position, and verbal encouragement was used with 1 min rest intervals between attempts. Relative muscle strength was calculated by the following formula: Relative muscle strength= [absolute muscle strength (kg)/body mass (kg)] (Prestes, 2013).

### 2.6. Visual analog scale (VAS) for pain

Pain intensity was evaluated by VAS for pain, which comprises a line, with extremities numbered from 0 to 10. In one extremity is marked “no pain” and in the other “worst pain imaginable.” Each subject freely pointed the pain present at that moment: Do you have pain? How do you classify your pain? According to Jensen *et al*. (2003):

- From 0 to 2, light pain;
- From 3 to 7, moderate pain;
- From 8 to 10, intense pain.

### 2.7. Quality of life

Quality of life was evaluated by the World Health Organization Quality of Life for the elderly (WHOQOL-OLD) questionnaire, which consists of 24 questions and its answers follow a Likert scale (from 1 to 5) attributed to six facets: “Sensory functioning” (SF), “AUT,” “past, present, and future (PPF) activities,” “social participation” (SP), “death and dying” (DD), and “intimacy” (INT). The “SF” facet evaluated the sensorial functioning and the impact of losing sensorial abilities on quality of life. The “AUT” facet referred to independence during aging and described at which point, it is capable to live with AUT and make own decisions. The facet “past, present, and future activities” described the satisfaction about life achievements and things to be reached. “SP” defined the participation in daily life activities, especially in the community. The “DD” facet was related to concerns, restlessness, and fears about death and to die, while “INT” facet evaluated the capacity to have personal and intimate relationships. The recodification of the scores followed the recommendations from [18].

### 2.8. Statistical analyses

The data are expressed as means±standard deviation (SD). Shapiro–Wilk tests were applied to check for normality distribution of the variables assessed. The internal consistency of the WHOQOL-OLD was assessed through Cronbach’s alpha coefficient. The facets were individually analyzed and a reliability coefficient was also determined for the set of 24 items. The WHOQOL-OLD module comprised 24 items recorded in a 5-point Likert scale, divided into six facets. Each facet consists of four items and thus generates independent scores ranging from 4 to 20 points (converted through syntax into a 0-100 scale). The six facets scores, combined with the answers of the 24 items, result in the overall score of the instrument. As for other WHOQOL instruments, higher scores represent better quality of life in the facets. Unpaired Student’s *t*-test was used to compare anthropometric, hemodynamic, strength, glucose, and quality of life scores between hypertensive and diabetic elderly. Correlations between quality of life scores and glucose, blood pressure, and strength were evaluated using Pearson product moment coefficients. The Cohen’s convention was used for assigning strength of association between the variables. The power of the sample size was determined using G*Power version 3.1.3, based on the difference of the level of pain between hypertensive and diabetic elderly. Considering the sample size of this study and an alpha error of 0.05, the power (1-β) achieved was 0.77 for the level of pain. The level of significance was *P*≤0.05 and SPSS version 20.0 (Somers, NY, USA) software was used.

## 3. Results

Table 1 presents the anthropometric and hemodynamic characteristics, muscular strength, and glycemia of elderly obese with hypertension or diabetes. Systolic (*P*<0.001) and diastolic (*P*=0.003) BP were statistically higher in elderly obese with hypertension as compared with elderly obese with diabetes. On the other hand, blood glucose concentration was significantly higher (*P*<0.001) in the diabetic group. No other anthropometric or strength differences (*P*≥0.05) were observed between the groups.

**Table 1:** Anthropometric and hemodynamic characteristics, muscular strength, and glycemia of elderly obese individuals with hypertension or diabetes (mean±SD).

	Hypertensive (*n*=35)	Diabetic (*n*=17)
Men, *n* (%)	12 (34%)	5 (29%)
Age, years	68.3±6.4	68.6±5.5
Height, cm	154.4±9.6	157.1±8.0
Weight, kg	79.1±13.7	77.9±10.1
Body mass index, kg/m^2^	33.1±4.9	33.7±8.8
Class I obesity, *n* (%)	26 (74.3%)	14 (82.4%)
Class II obesity, *n* (%)	6 (17.1%)	3 (17.6%)
Class III obesity, *n* (%)	3 (8.6%)	-
Waist circumference, cm	98.7±28.2	99.6±25.7
Waist-to-hip ratio	1.07±0.30	1.03±0.26
Glycemia, mg/dL	134.7±36.2	216.9±55.8*
Systolic BP, mmHg	150.6±17.8	131.2±9.3*
Diastolic BP, mmHg	89.2±11.3	79.4±8.3*
Palmar grip strength, kg/f	32.7±10.2	30.9±12.4
Relative muscular strength	0.41±0.11	0.38±0.12

BP: Blood pressure;

* *P*<0.05 between groups

**Figure 1:**
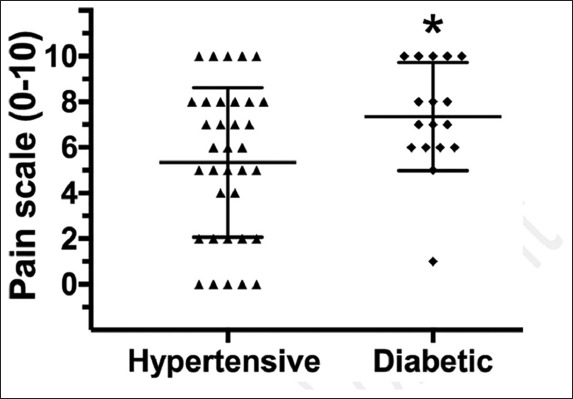
Pain scale (0-10 points) reported by elderly obese with hypertension or diabetes (mean±SD). *P≤0.05 versus the hypertensive group.

The level of pain reported by hypertensive elderly obese (5.3±3.4) was significantly lower (*P*=0.026) than the level of pain reported by the diabetic elderly obese (7.4±2.4; Figure 1). Within the hypertensive group, 29% classified the pain as light, 40% as moderate, and 31% as intense. For the diabetic group, 6% presented a light pain, 47% moderate, and 47% intense.

The internal consistency of the WHOQOL-OLD, assessed through Cronbach’s alpha coefficient, is presented in Table 2. The Cronbach’s alpha coefficient values revealed an excellent internal consistency to the 24 questions together and to the facets of SF, death and die, and INT. Internal consistency was considered good for the six facets together, AUT, past, present, and future activities and SP.

**Table 2:** Cronbach’s alpha coefficient for the World Health Organization Quality of Life facets and questions (*n*=52).

	CC	NI
Facets	0.856	6
24 questions	0.952	24
Sensory abilities	0.929	4
Autonomy	0.882	4
Past, present, and future activities	0.853	4
Social participation	0.828	4
Dead and dying	0.956	4
Intimacy	0.976	4

CC: Cronbach’s alpha coefficient, NI: number of items.

The general score of quality of life did not present statistical differences (*P*=0.097) between the hypertensive and diabetic groups. However, the scores for the facets of SF (*P*=0.029) and past, present, and future activities (*P*=0.043) were significantly higher for elderly obese with hypertension when compared to elderly obese with diabetes. No other differences (*P*≥0.05) were observed between the groups with respect to the other WHOQOL-OLD facets (Table 3). No statistically significant correlations (*P*>0.05) were observed between scores for the quality of life and glucose, or blood pressure and muscular strength. Sex, age, obesity classification, and illness time also did not exhibit statistically significant correlations between groups.

**Table 3:** Quality of life scores in general and for specific facets in elderly obese with hypertension or diabetes (mean±SD).

	Hypertensive	Diabetic
General	78.2±18.2	69.8±18.9
Sensory functioning	13.1±4.0	11.1±4.3^*^
Autonomy	13.1±4.6	11.1±3.4
Pass, present, and future activities	13.5±3.0	12.4±2.9^*^
Social participation	14.0±2.8	12.5±2.7
Dead and dying	12.5±4.6	11.2±4.2
Intimacy	11.9±5.0	11.4±5.7

* *P*≤0.05 as compared to the hypertensive group

## 4. Discussion

The aim of the present study was to compare pain levels, muscle strength, and quality of life in elderly obese individuals with diabetes or hypertension. The initial hypothesis was confirmed as the results revealed that elderly obese with diabetes presented higher levels of pain and worse scores in the sensory, past, present, and futures activities facets of quality of life. In addition, there were no differences in handgrip strength or any other facets of quality of life.

Through health programming, such as the Hiperdia program, in which elderly participants in the current study were enrolled, care for different diseases can be improved by increasing knowledge of specific particularities, such as higher pain levels and worse quality of life. As mentioned earlier, most studies focused in comparing elderly subjects with or without a disease, and there is a gap of investigation between diseases. The sample in the present study was composed predominantly of women, with 64% of elderly obese with hypertension group being female, and 71% of elderly obese with diabetes, which reinforces evidence that women search out and participate in health services to a greater extent than men [9]. In this sense, future studies should also focus on elderly male population with these chronic diseases.

There were no differences between elderly obese with hypertension or diabetes in anthropometric variables or handgrip muscle strength. Tibana *et al*. [19] investigated young sedentary women and revealed that women with higher handgrip strength presented lower BMI as compared with women with lower muscle strength. Another study from Teixeira *et al*. [20] found that overweight/obese middle-aged women had lower muscle strength as compared with eutrophic women [20]. According to Schrager *et al*. and Maltais *et al.*, obesity alone, particularly central adiposity, increases pro-inflammatory cytokines resulting in protein catabolism, loss of muscle mass, and decreases in muscle strength. Thus, results of the present study revealed that the combination of the aging process and obesity was sufficient to decrease muscle strength and modify body composition, without additional effects of diabetes or hypertension.

The benefits of exercise and resistance training have been widely demonstrated, including vascular protection, and a decrease in blood pressure and increase in the bioavailability of nitric oxide, which is an important vasodilator [19,21,22]. Bottcher *et al*. [23] compared the physical fitness of hypertensive and normotensive subjects and found that hypertensive subjects had decreased scores of muscle strength, cardiorespiratory fitness, balance, agility, coordination, and flexibility as compared with normotensive subjects. Moreover, 6 months of exercise training did not decrease the differences between groups [23]. It is difficult to precisely determine if hypertension decreases physical fitness or whether lower scores of these variables causes hypertension.

Deshmukh *et al*. (2017) reported that type 2 diabetes patients that were successful in controlling the disease presented lower BMI compared to patients who were not stable [24]. In patients with uncontrolled diabetes (*n*=25), manifestations of musculoskeletal pain were reported in 80% (*n*=20) [24]. Alterations due to diabetes contributed to reduced hand and shoulder mobility and increased weakness of the ankle and knee joints, which may decrease physical capacity [25,26]. A review study from Oliveira *et al*. revealed that subjects with diabetes present decreased handgrip strength [27]. Savas *et al*. (2007) also found lower handgrip strength, and more pronounced hand dysfunctions in the elderly with diabetes as compared with the elderly without diabetes [28]. As mentioned earlier, the results of the present study highlight the importance of comparing elderly individuals with distinct diseases, as opposed to those with and without a particular condition. Results from the current study revealed that elderly obese with diabetes or hypertension had no differences in muscle strength.

The level of pain reported by elderly obese individuals with diabetes was higher as compared with elderly obese with hypertension. This result corroborates with findings from Papanas and Ziegler *et al.*, which reported an elevated incidence of pain in neuropathic diabetes [29]. In this case, the results of the present study revealed that diabetes was a primary determinant to increased pain sensation, even in the presence of aging, obesity, and hypertension. This should be considered as an important clinical feature in the treatment and health care of elderly individuals with diabetes.

Feelings of pain were evaluated by the VAS and results indicated that among elderly obese individuals with diabetes, 6% had light pain, 47% moderate pain, and 47% intense pain. Moreira *et al*. [30] used the same pain scale to evaluate distal polyneuropathic pain of the lower limbs in 204 patients with type 2 diabetes. The study revealed that moderate pain was present in 37% of the patients, severe pain in 40%, and light pain in 3%. Pedras *et al*. [31] also found a higher prevalence of maximum pain followed by moderate and minimal pain in 206 elderly with type 2 diabetes and diabetic foot ulcer indicative of amputation surgery. With regard to pain associated with hypertension, Carvalho *et al*. reported lower levels of pain in hypertensive patients, which appears in the event of an additional cardiovascular complication, such as myocardial infarction [32].

It should be noted that the present study did not investigate specific sites of pain, which may influence the results [33]. It has been demonstrated that hypertensive subjects tend to have pain in the head, neck, chest, and lower limb, while subjects with diabetes reported pain more frequently in the lower limbs and in the head [34]. A study from Dellaroza *et al*. revealed that subjects with hypertension had higher percentage of chronic pain (66.5%) as compared with subjects with diabetes (23%) [35]. However, the discrepancy in these results may be related to the prevalence of other chronic diseases in the population studied, such as cataracts, osteoporosis, and arthritis. Thus, based on the available literature to date, this area seems to be a promising field of study in elderly individuals with different diseases.

In general, quality of life was not different between elderly obese individuals with hypertension or those with diabetes. It is important to mention that the elderly are resilient to disease problems and symptoms because they typically are accustomed to facing difficulties throughout the lifespan and, in some cases, can count on good support from their family [36]. However, with respect to the facets of SF and PPF activities, elderly obese subjects with diabetes presented lower scores as compared to elderly obese with hypertension. It is possible that dependence on health services and family caused by diabetes complications could result in lower quality of life scores in some facets [14]. Considering that the SF facet investigates the impact of the loss of senses on quality of life [18], the reduction in this facet could be related to diabetes-induced chronic renal failure, lower limb amputation, blindness, cardiovascular diseases, and other mechanisms [37]. Moreover, it is difficult for subjects with diabetes to adapt to a restricted diet, medications, and deleterious effects of the disease in the general system [14,38], which may also be related to the lower scores in the PPF activities facet observed in the present study.

Among the possible limitations of the present study are the cohort size (despite adequate sample power) and the fact that all the elderly were obese and sedentary, which may limit the interpretation of these results to these specific populations. Future investigations should aim to include physically active and trained elderly individuals, as well as analyze the sites of pain and main causes.

## 5. Conclusion

Elderly obese patients with hypertension or diabetes present different SF and PPF activities facet of quality of life, while no differences were observed in the other facets. Elderly obese subjects with diabetes also reported more pain than elderly obese individuals with hypertension. With the increasing demand of elderly population, the knowledge of specific features of each disease during the aging process is important to orient the treatment and management of these patients.
